# Benthic baselines of the Groote Archipelago’s key reefs in the wake of mass coral bleaching and Tropical Cyclone Megan

**DOI:** 10.1371/journal.pone.0353017

**Published:** 2026-08-12

**Authors:** Dane P. G. Wattle, Ariel K. Pezner, Annabelle Doheny, Hadley England, Anindilyakwa Land and Sea Rangers, Emma F. Camp

**Affiliations:** 1 Climate Change Cluster, University of Technology Sydney, Ultimo, NSW, Australia; 2 Anindilyakwa Land and Sea Rangers, Groote Eylandt, NT, Australia; Secretariat of the Pacific Community, NEW CALEDONIA

## Abstract

Coral reefs around the world are experiencing increasingly frequent and intense stress events, leading to benthic phase shifts. The ecological consequences of this are often profound and lasting, necessitating the development of both effective and regionally specific conservation strategies. Actualising positive conservational outcomes require managers to first gain insight into the present ecosystem diversity and structure; yet, in Groote Eylandt, a remote reef and largest island in the Gulf of Carpentaria off the northern end of Australia, this has not occurred. The Traditional Owners of Groote Eylandt (the Anindilyakwa people) have a long history of connection to the sea, but despite this rich history, they have not formally documented changes to their reef systems, which can create challenges for modern collaborative management efforts. Consequently, this study aims to document the benthic composition of key reef sites within the Groote Archipelago. Considering recent consecutive stress events, in the form of coral bleaching and Tropical Cyclone Megan, assessments of individual site health were also performed through frame compositional analysis of video surveys captured using a remotely operated vehicle. Benthic composition of five reefs within the Groote Archipelago were successfully documented. Scleractinian coral cover was documented at all sites, ranging from 5.2–47.1%, although dominant morphology and levels of mortality were site-specific. Branching morphologies were exceedingly rare, with coral composition dominated by massive morphology corals at the sites surveyed. Other major benthic taxa included soft corals, macroalgae, and sponges. Sea surface temperature data from satellites revealed that temperatures were elevated prior to bleaching observations but did not exceed a Degree Heating Week of 2.1, placing these reefs into the “Bleaching Warning” category. Our findings establish a foundational understanding of benthic composition at Groote Eylandt, necessary for further marine conservation within the region. This work also highlights the importance of acquiring data at remote reefs where data deficiencies can complicate local management efforts.

## Introduction

Increasingly frequent and intense stress events are instigating phase-shifts within marine benthic communities worldwide [1]. These shifts, typically evident within coral reefs through spatial and temporal changes in dominant benthic composition, are induced through a combination of biotic (*e.g.*, competition or predation) and abiotic (*e.g.*, temperature, eutrophication, or wave exposure) pressures [1–4]. Globally, coral reefs are trending towards degradation [5] and, in some places, far more rapidly than previously anticipated [1–4]. As suitable reef habitat becomes increasingly fragmented, loss of vulnerable species can have serious consequences for local biodiversity [6,7]. Such processes are well documented within many nationally and internationally significant reefs (*e.g.*, the Great Barrier Reef and Ningaloo Reef in Australia) [8,9]. However, effects within less well-known or easy to access reefs may be obscured by a lack of historical baselines in published scientific literature [10] – though these reefs are no less important to local ecosystems and peoples. Thus, providing accurate and assessable baselines of ecosystem health for remote or under-studied reefs has become increasingly important to ensure optimal and equitable conservation efforts across diverse reef systems [11].

As a representation of a reef’s structure, benthic analysis is commonly utilised to assess ecosystem baselines [12]. Understanding benthic composition is imperative to assessing overall reef functioning [13], as features such as substrate class and structural complexity (*e.g.*, rugosity), which is influenced by the variety of coral morphologies (branching, massive, foliose, etc.) on a reef, holds considerable influence over biotic assemblages and reef functioning [14]. Crucially, the sessile nature of major reef benthic inhabitants allows for long-term study of changes in composition over space and time [11,15]. The relative abundance and morphological diversity of the benthos supports other diverse marine taxa; availability of preferred substrate can impact the growth and settlement of sessile recruits, and motile taxa may rely on sufficient benthic rugosity for shelter [16,17]. Broadly, benthic substrate may be categorised as consolidated (hard) or unconsolidated (soft), though geological composition and contiguous area are equally important distinctions [16,17]. Maintaining sufficient structural diversity is a key driver of reef health and biodiversity [16,17]. Thus, benthic compositional changes may lead to or be indicators of a decline in reef functioning and health [18].

Methodologies for assessing benthic composition range from *in situ* visual snorkelling transects, to *ex situ* video analysis, and sonar-detected rugosity [19–21], depending on project scope and setting, as well as desired accuracy, speed, and budget [22]. For reefs that are deep or otherwise inaccessible to divers, remotely operated vehicle (ROV) surveys with attached cameras are a popular choice [23,24]. This method is especially useful when working in hazardous waters (like those around Groote Eylandt in Australia, due to the presence of crocodiles) [23], while still capturing data at appropriate spatial scales [25,26]. To deal with the large number of images captured during such surveys [27], real-time segmentation models can provide researchers with the necessary tools for rapid and systematic compositional analysis of frames, while still maintaining enough granularity to assign objects in the videos to useful categories for analysis [28]. Groote Eylandt in the Gulf of Carpentaria, like many Northern Territory reefs of Australia, is both understudied and poorly understood by the scientific community (outside of the Traditional Owners of this region). Living coral reefs in the Gulf of Carpentaria were only noted in published scientific literature in 2004 [29] and further research has revealed that the Gulf of Carpentaria is home to an expansive reef system [30]. No official estimate of reef size currently exists, though satellite imagery indicates the extent to be > 3,200 km^2^ [31]. Concerningly, only seven papers pertaining to coral reefs in this region have been published in the twenty years since the initial mention of these reefs in the scientific literature (S1 Fig). Of these, none were conducted around the region’s largest island, Groote Eylandt; nor did any concern the titular component of reefs: corals. Given the immense ecological importance of corals, resolving knowledge gaps on coral diversity and abundance will be key for regional conservation efforts. Outside of published scientific literature, the Traditional Custodians of Groote Eylandt, the Anindilyakwa people, hold significant knowledge of local reef systems and have substantial connection to reef health, production, and conservation [32,33]. Importantly, local communities around Groote Eylandt rely on healthy marine systems for hunting and sustenance [33]; however, like many reefs globally, the ongoing survival of Groote Eylandt’s reefs is threatened by rising ocean temperatures [1,5], cyclones [34], and other local stressors such as changes to water quality [35].

Groote Eylandt is also facing threats from climate change, through mass coral bleaching events; a wide-spread event where corals lose their symbiotic algae because of environmental stress [36]. In February 2024, a bleaching event affecting numerous coral colonies around Groote Eylandt was observed (per obs. A. Doheny), resulting in full or partial bleaching. Subsequent cyclonic activity likely damaged reef structures further (Tropical Cyclone Megan, 16^th^ – 18^th^ March 2024; category 4), though the consequent influx of cold-water may have alleviated thermal stress [37]. Given the lack of existing data on biodiversity in this area, the impacts of these back-to-back stress events on local reef biodiversity is not known [38,39]. Thus, establishing the present-day health and benthic composition of these local reefs is fundamental to developing effective conservation plans together with the local ecological knowledge of the Anindilyakwa people [40].

Collecting present-day benthic condition data, with site selection guided by Traditional Ecological Knowledge, is key for determining reef trajectory at understudied locations like Groote Eylandt [41]. In this study, we establish quantitative baselines of benthic composition at 5 key reefs of the Groote Archipelago, as determined by the Anindilyakwa people, through benthic surveying [42]. Considering the true baselines of these ecosystems may be obscured by recent bleaching and cyclone damage, these surveys tangentially aim to estimate site variation in coral degradation – visible as either mortality or rubble. We also investigate trends in local sea surface temperature prior to observed bleaching to understand if temperature alone can explain the bleaching patterns observed in the field. Results from this study are timely and will have direct application towards the creation of regional conservation strategies and a Sea Country Management Plan in partnership with the Anindilyakwa people.

## Methods

### Study Sites

**Fig 1:**
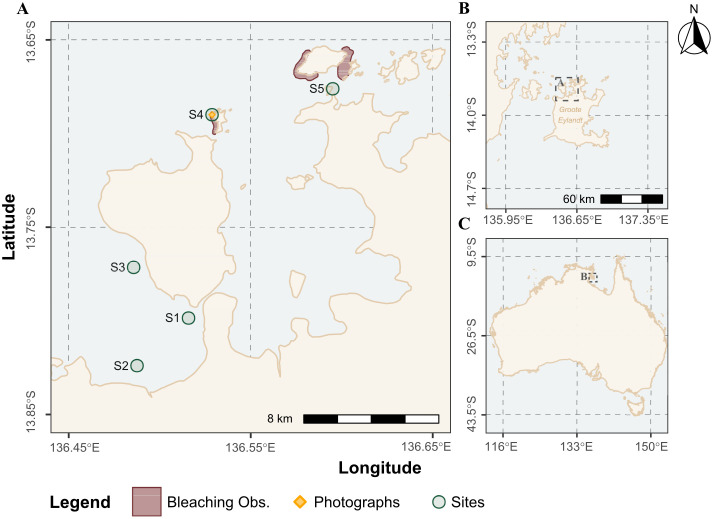
Map of study sites and bleaching observations within the Groote Archipelago, Australia. (A) Site locations are shown as green circles with their corresponding number displayed adjacently. Range of February 2024 bleaching observations (obs.; Sites 4 and 5 only) are highlighted by red shading, with the site of contemporaneous photographs denoted by a concentric yellow diamond. (B) Overview map depicting the location of study sites (dashed square) relative to Groote Eylandt and (C) the wider Archipelago. Map data was sourced from the gadm function in the ‘geodata’ package in R.

The Groote Archipelago is located within the north-west of the Gulf of Carpentaria (Fig 1). This region is relatively shallow (<70 m deep), with waters along the island’s north and west coasts rarely exceeding 20 m in depth [43]. Tropical cyclones are a frequent occurrence within the region [43], as demonstrated by the recent Cyclone Megan (16^th^ – 18^th^ March 2024). Long-term manganese mining operations have also taken place on Groote Eylandt since 1965 [44]. Consequently, much of the waters off Groote Eylandt suffer from excessive turbidity and sedimentation [45]. Water cycling within the basin can take upwards of two years, resulting in strong thermal stratification and stagnation of water flow [46]. These features combined make Groote’s reefs likely to be what we could consider ‘marginal reefs’, due to the perceived ‘sub-optimal’ conditions in the area [47]. Saltwater crocodiles (*Crocodylus porosus*) and box jellyfish (Cubozoa) are commonly seen in these reefs [48,49], posing challenges to any fieldwork.

As this study has been co-designed with the Anindilyakwa people from inception, site locations were chosen in accordance with their needs and level of cultural importance. The Anindilyakwa are intimately connected with their Sea Country, with their knowledge of reef locations and composition dictating locations for benthic surveying efforts [33]. This process began with an initial meeting between E. Camp, H. England, and Elders in September 2023 to discuss community knowledge and interest, and subsequent agreement of project scope. Five sites from the Archipelago’s north were surveyed from 2^nd^ - 5^th^ June 2024, two of which (Sites 4 and 5) had previously been visited by the Anindilyakwa Land and Sea (ALS) Rangers in February 2024 during the mass bleaching event (Fig 1). All research was approved by the Anindilyakwa Land Council (no permit numbers are issued in this region) and covered by Northern Territory Fisheries permit S17/3590.

### Bleaching Surveys

Widespread bleaching of coral colonies was observed on 11^th^ February 2024 by the ALS Rangers during routine fieldwork. While not equipped to undertake rigorous surveys, opportunistic photographs were taken to document the extent and severity of bleaching. Bleaching was observed off the coasts of Chasm (Site 5) and Little Winchelsea Islands (Site 4), with all photographs captured at the latter (Fig 1). Visual analysis of photographs was undertaken *ex situ*, with colonies assigned colour categories using the *CoralWatch* bleaching index [50].

### Benthic Surveys

Benthic surveys were conducted by the team from University of Technology Sydney (UTS) and the ALS Rangers from 2^nd^ June – 5^th^ June 2024 at locations selected by the ALS Rangers based on their knowledge of the region. Saltwater crocodile activity poses risks to any fieldwork in the region and as such, manual transects were impossible. Instead, surveys employed a remote operated vehicle (ROV; *BlueROV2*, Blue Robotics, California, USA) to safely complete transects. Cameras (GoPro Hero 11, Go Pro Inc., California, USA) were affixed to the ROV undercarriage to capture high-resolution footage of the benthos. The ROV was subsequently deployed off the Miyabunja II and Makarda II vessels. Surveys employed a transect-like approach, capturing continuous footage of the benthos in a sweeping arc with “repetitions” achieved by frame sub-sampling. Due to logistical constraints, survey times varied between sites (22.5 to 32.5 minutes). Furthermore, inconsistent site topography impacted ROV ability to capture videos at an ideal distance from the benthos (*i.e.*, 2 m), though all sites had a depth range of 1–5 m. Resulting surveys ranged from 0.2 to 4.0 metres from the benthos, necessitating an adjusted approach to video analysis to standardise sampling between sites (see below). Consequently, effective site dimensions varied and were dependent on the number of useable frames recoverable.

### Image Processing for Benthic Surveys

Image processing combined high-throughput methods with manual validation techniques. Footage gathered from each site was converted into JPEG still-frames using FFmpeg [51] at 1 frame per second. ROV velocity was often non-linear, requiring manual subsampling of still-frames to eliminate individual overlap. Each frame was further assessed for clarity, with those with significant obstruction (*e.g.*, bubbles, excessive turbidity, or the ROV tether) or outside accepted distance range (0.3–3.0 m from the benthos; extended from 2 m as necessitated by ROV buoyancy difficulty) removed, culminating in 1000 usable frames across all sites. Images were subsequently uploaded to BIIGLE 2.0 (BioImage Indexing, Graphical Labelling and Exploration) [52] for annotation and segmentation (S2 Fig). BIIGLE is an online portal providing several tools to assist with high-throughput annotation of marine imagery, from Machine Learning Assisted Image Annotation (MAIA) for the automated detection of objects of interest [53] to a Segment Anything vision Model (Magic SAM) [28]. This study utilised the latter to quickly and effectively perform whole-image segmentation, adjusting manually as required. Frames were assigned distance values simultaneously based on visual assessment benthos-camera distance. This information was used to generate real-word area calculations of image footprints and annotations using the DELPHI (DEtection of Laser Points in Huge image collection using Iterative learning) tool [54]. Benthos-camera distance and corresponding area calculations were verified for each category using the BIIGLE measurement tool on objects of approximately known size (*i.e.*, *Acropora* spp. branchlet widths as determined from supplemental collections for another study).

To facilitate cross-usability of data, annotations used the expert-constructed and internationally recognised CATAMI (Collaborative and Annotation Tools for Analysis of Marine Imagery) classification scheme [42]. While only capable of identifying taxa at high level, this scheme is adequate given that delineation of coral to species-level from benthic imagery is challenging [55]. Segmentations were annotated to the lowest CATAMI sub-category possible, systematically working backwards through parent categories until certainty was achieved. Scleractinian coral mortality was the only exception, being broadly assigned as “Corals> Stony Corals” supplemented with the “Dead” qualifier as further distinction was largely impossible. To counteract site-footage variance, annotation area of each label type was summed per site and standardised as a proportion of their total annotated area. Corals were assessed contemporaneously for signs of colony-bleaching, determined visually by colour saturation.

### Sea Surface Temperature

Nightly Sea Surface Temperature (SST) readings were obtained for all sites except Site 1 from the National Oceanic and Atmospheric Administration (NOAA) *CoralTemp* v3.1 global 5 km Satellite [56]. Discrete readings for Site 1 were unobtainable due to the nearest *CoralTemp* resolution node falling over land. The next closest belonged to Site 2, thus Sites 1 and 2 were grouped accordingly. Daily data was downloaded from 1st June 2023 – 31st May 2024 to capture the summer before bleaching and any persisting effect until fieldwork began (1st June 2024). Historic SST range and calendar date means were drawn from the twenty years prior to the bleaching event (*i.e.*, June 2003 – May 2023). Site bleaching thresholds (*sensu* Skirving et al. [56]), defined as the temperature above which thermal stress is typically suggested to begin, were used to identify periods of severe thermal stress [56]. Thresholds were defined for each site as +1.0 °C above their maximum monthly mean (MMM) SST, the mean temperature of the historically warmest month [56]. The severity of thermal stress at each site was determined using Degree Heating Week (DHW) values, calculated as the number of days and degrees Celsius that bleaching thresholds were exceeded over any given 12-week period [56].

### Statistical Analysis

All data analysis and visualisation was conducted in RStudio (v4.3.0) [57]. Functions utilised during analyses were drawn from the packages: *stats* (base R), *vegan* [58], *FSA* [59], and *indicspecies* [60]. All graphs were generated using the package *ggplot2* [61]. Data was checked for normality and relevant assumptions prior to analysis, with nonparametric alternatives being conducted as required. Having satisfied these assumptions, a series of one and two-way ANOVAs (Analysis of Variance; *stats::aov* function) were employed to determine the site, year, seasonal effects on SST data, including any interaction therein. Individual drivers within each effect were discerned through pairwise post-hoc analyses (Tukey’s Honestly Significant Difference; *stats::TukeyHSD*). Benthic compositional data, however, failed assumptions of normality. To counteract site-footage variation, annotation area of each label type was summed per site and standardised as a proportion of total annotated area. As ROV mechanical failure precluded traditional transect analysis, a quadrat-like approach was utilised for all statistical test repetitions (*i.e.*, individual frames). While not ideal, the lack of inter-frame overlap facilitated their use for this purpose. Site differences in mean proportional cover for each CATAMI classification were assessed through Kruskal-Wallis H tests (*stats::kruskal.test*), with post-hoc pairwise tests performed using Dunn’s Multiple Comparisons (*FSA::dunnTest*) with false discovery rate correction per Benjamini and Yekutieli [62]. Frame benthic-compositions were converted into Bray-Curtis dissimilarity matrices, with site clusters visualised through non-metric multidimensional scaling (nMDS; *vegan::metaMDS*). nMDS-derived ordination was selected from the lowest-stress model of >500 random initialisations (k = 2), further repeated until no improvement was evident within 100 consecutive attempts. Drivers of ordination shape were determined through vector analysis of benthic categories and frame ‘environmental’ variables (*i.e.*, distance to benthos and total area), as calculated by permutational multiple regression (*vegan::envfit*). Multivariate analysis of benthic composition between sites was guided by permutation dispersion analysis (PERMDISP; *vegan::betadisper*), with variation between sites ultimately assessed using analysis of similarities (ANOSIM; *vegan::anosim*). Pairwise comparisons of site benthic dissimilarity and benthic features driving these dissimilarities were determined through post-hoc similarity percentage analysis (SIMPER; *vegan::simper*). Finally, to identify which benthic classifications were indicators of any given site (or combination thereof) and assist the ALS Rangers with ongoing Sea Country management, indicator species analysis (ISA; *indicspecies::multipatt*) was conducted to provide information about site-similarity and/or uniqueness in a more digestible format to prioritise certain sites for future management or study. All permutational analyses were conducted using a set seed (n = 1) and assessed sequentially up to 9999 permutations.

## Results

### Bleaching Surveys

**Fig 2:**
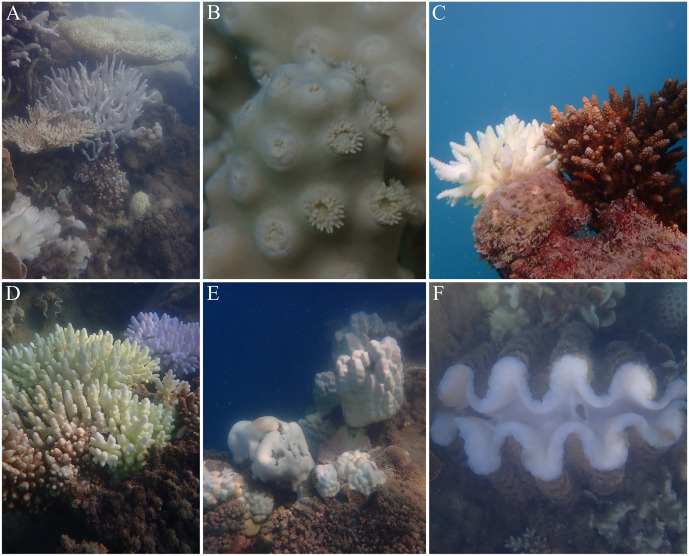
*In situ* photographs of the 2024 mass bleaching event around Little Winchelsea Island within the Groote Archipelago. Scleractinian corals of various bleaching severity are depicted, ranging from fluorescence to outright mortality, captured on 11 Feb 2024. Various morphotypes were impacted, highlighted by bleaching of: (A) branching and tabulate; (A, B, E) sub-massive; (A, E, F) massive; (A, C, D, F) corymbose; and (A, F) foliose corals. (F) Contemporaneous bleaching of clams (*Tridacna* spp.) within the same locality was also observed. Mosaic compiled using Biorender.com.

A total of 67 photographs were collected on 11th February 2024, capturing varying degrees of coral colony bleaching (Fig 2). All coral morphologies were impacted to varying extents (see Fig 2A-F), ranging from fluoresced (a sign of stress) to deceased. Event impact was not isolated to Scleractinia; other marine taxa, including giant clams (*Tridacna* spp.), also exhibited visible bleaching (see Fig 2F). Corals were observed at 1–6 m depth, with most displaying colony-wide bleaching. Indeed, photo analysis revealed 62.3% of colonies to be either category one or two under the Coral Watch bleaching index (the most severe levels).

### Benthic Surveys

#### Survey Coverage.

**Fig 3:**
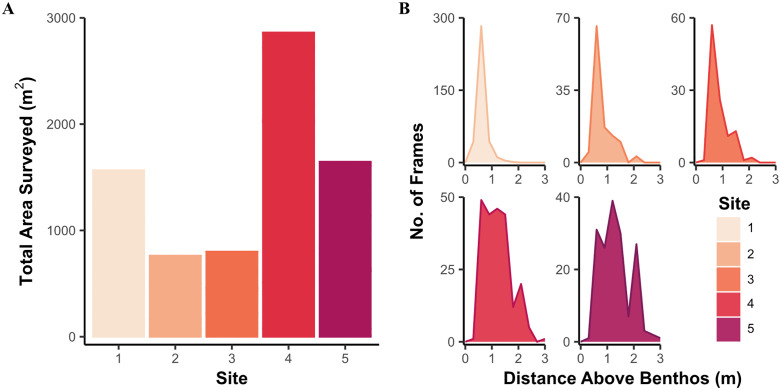
Summary of usable video frames from ROV benthic surveys at five sites within the Groote Archipelago. (A) Total frame-area of each site used within study analyses. (B) Number of usable image frames per site following processing by estimated distance from benthos. Note that plots in Panel B use varying y-axis scales to better show the distribution of distance above benthos for the frames captured at each site.

**Fig 4:**
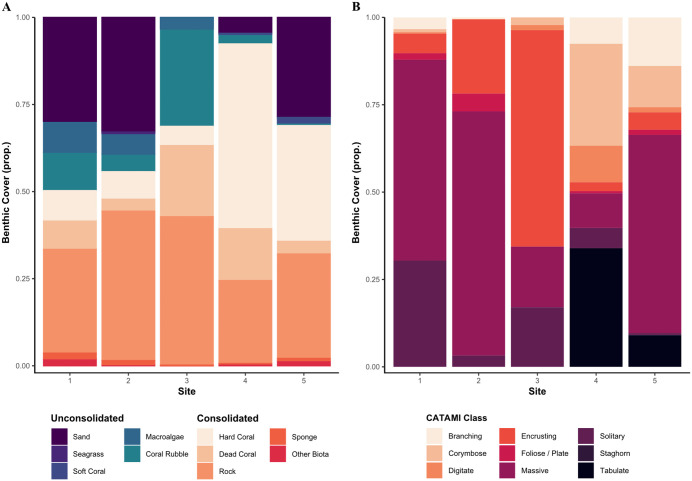
Proportional benthic composition of five reefs within the Groote Archipelago. (A) Comprehensive benthic classifications of each site, with physical consolidation broadly categorised by hue (blues, unconsolidated; reds, consolidated) and putative ecological function categorised by individual colour. Labels are indicative of CATAMI parent classifications, except “Hard Coral”, which includes both *Heliopora* spp. (Black & Octocorals) and scleractinian corals (Stony Corals) due to their similarly reef-building nature. All other Black & Octocorals are classified under “Soft Coral”. (B) Proportional composition of scleractinian coral morphologies within each site, defined as all CATAMI sub-classifications of “Corals> Stony Corals”).

Benthic surveys conducted from 2^nd^ June – 5^th^ June 2024 resulted in 1000 usable frames across all sites. Following frame distance-area calculations, surveyed area totalled 7,635.7 m^2^ (Fig 3A). Area-share was disproportionate between sites, heavily favouring Site 4 (2,861.5 m^2^). Poor visibility at Sites 2–3 resulted in the lowest area coverage of all sites (762.1 m^2^ and 800.0 m^2^, respectively). Similarly, most frames from these sites were <1 m from the benthos (Fig 3B). Annotation labels drew from 59 unique CATAMI classifications. After aggregating into parent classifications where necessary (*e.g.*, sub-feature uncertainty within some sites), benthic analyses utilised 46 of these classifications. All classifications could be subcategorised into 10 distinct categories, and further distinguished into “unconsolidated” (sand, seagrass, soft coral, macroalgae, and coral rubble) and “consolidated” (hard coral, dead coral, rock, sponge, and other biota) substrate (Fig 4A).

#### Benthic Composition.

All sites surveyed were predominantly consolidated in nature (Fig 4A), ranging from 50.3% (Site 1) to 92.4% (Site 4). Bedrock was the largest single classification by area in all sites, though inter-site variation was apparent (H = 33.579, p < 0.0001; Kruskal-Wallis; Table 1). Post-hoc analysis revealed Site 3 to have significantly more consolidated substrate (inclusive of bedrock, boulders, and cobble classifiers) than other sites (41.4%; Dunn’s; Table 1). Unconsolidated sand had inconsistent detection between sites, with cover differing significantly between two site clusters: Sites 3 and 4; and Sites 1, 2, and 5 (Kruskal-Wallis and Dunn’s; Table 1). No fine sand or silt was detected at any site; all sand contained considerable biogenic content. Conversely, hard coral cover was detected at all sites, occupying as much as 52.7% of the benthos at Site 4. Dead coral was present at all sites, making up 20.8% of overall benthic composition at Site 3 and 14.7% at Site 4. However, percent coral mortality (*i.e.*, percentage of dead coral cover relative to total coral cover) was greatest at Site 1 (50.2%) and Site 3 (80.0%). Similarly, these sites exhibited the greatest percentage of coral rubble cover relative to overall benthic composition (11.0% and 27.9%, respectively; Dunn’s; Table 1). Coral assemblages at Site 5 were comparatively healthier, only exhibiting 2.7% coral rubble cover and 4.0% dead coral cover (a percent mortality of 10.8%). Accordingly, all coral mortality metrics were significantly lower at Site 5 than at Sites 3 and 4, though the latter two did not significantly differ from each other (Dunn’s; Table 1). No whole-colony bleaching was detected during benthic surveys within any site.

**Table 1:** Kruskal-Wallis H statistic and corresponding p-value for mean frame benthic composition of each study site.

CATAMI Classification		Overall		Dunn’s Multiple Comparison (Z-score \ p-value)					
Parent	Class	H-stat	p-value		Site 1	Site 2	Site 3	Site 4	Site 5
**Stony Corals**	> Branching	135.91	<0.001	**Site 1**	–	1.000	0.417	0.000	0.004
				**Site 2**	0.913	–	1.000	0.000	0.004
				**Site 3**	1.581	0.547	–	0.000	0.001
				**Site 4**	−10.072	−8.208	−8.763	–	0.000
				**Site 5**	−3.324	−3.335	−3.905	5.277	–
	> Corymbose	416.91	<0.001	**Site 1**	–	1.000	0.768	0.000	0.000
				**Site 2**	0.105	–	0.887	0.000	0.000
				**Site 3**	−1.254	−1.097	–	0.000	0.000
				**Site 4**	−18.440	−13.577	−12.199	–	0.000
				**Site 5**	−9.954	−7.681	−6.426	6.163	–
	> Dead	130.98	<0.001	**Site 1**	–	0.001	0.000	0.000	0.001
				**Site 2**	3.594	–	0.000	0.000	1.000
				**Site 3**	−5.256	−7.118	–	1.000	0.000
				**Site 4**	−6.385	−7.992	0.243	–	0.000
				**Site 5**	3.811	−0.248	7.505	8.696	–
	> Digitate	259.21	<0.001	**Site 1**	–	1.000	1.000	0.000	0.078
				**Site 2**	0.382	–	1.000	0.000	0.128
				**Site 3**	0.087	−0.235	–	0.000	0.215
				**Site 4**	−14.826	−11.192	−10.824	–	0.000
				**Site 5**	−2.475	−2.222	−1.948	9.954	–
	> Encrusting	59.876	<0.001	**Site 1**	–	0.000	0.000	0.000	0.000
				**Site 2**	−4.215	–	0.445	1.000	1.000
				**Site 3**	−6.263	−1.689	–	0.054	0.544
				**Site 4**	−4.411	0.675	2.607	–	0.918
				**Site 5**	−5.282	−0.328	1.514	−1.149	–
	> Foliose/ Plate	50.709	<0.001	**Site 1**	–	1.000	1.000	0.023	0.000
				**Site 2**	−0.506	–	1.000	0.420	0.000
				**Site 3**	0.671	0.947	–	0.032	0.000
				**Site 4**	−2.889	−1.644	−2.715	–	0.004
				**Site 5**	−6.459	−4.480	−5.476	−3.465	–
	> Massive	180.08	<0.001	**Site 1**	–	1.000	0.000	1.000	0.000
				**Site 2**	0.982	–	0.005	1.000	0.000
				**Site 3**	4.976	3.234	–	0.000	0.000
				**Site 4**	0.139	−0.807	−4.510	–	0.000
				**Site 5**	−10.382	−8.777	−12.228	−9.501	–
	> Solitary	92.130	<0.001	**Site 1**	–	0.000	1.000	0.261	0.000
				**Site 2**	5.006	–	0.000	0.000	1.000
				**Site 3**	−0.685	−4.555	–	1.000	0.000
				**Site 4**	−1.864	−5.994	−0.716	–	0.000
				**Site 5**	6.956	0.911	5.863	7.822	–
	> Staghorn	10.651	0.031	**Site 1**	–	1.000	1.000	1.000	0.062
				**Site 2**	0.000	–	1.000	1.000	0.280
				**Site 3**	0.000	0.000	–	1.000	0.196
				**Site 4**	−0.844	−0.617	−0.612	–	0.271
				**Site 5**	−3.074	−2.344	−2.325	−2.086	–
–	> Sub-massive	139.61	<0.001	**Site 1**	–	1.000	0.026	0.000	0.176
				**Site 2**	0.169	–	0.144	0.000	0.318
				**Site 3**	2.785	2.115	–	0.000	0.000
				**Site 4**	−9.987	−7.457	−9.817	–	0.000
				**Site 5**	−1.977	−1.656	−3.945	6.425	–
	> Tabulate	350.29	<0.001	**Site 1**	–	1.000	1.000	0.000	0.000
				**Site 2**	0.000	–	1.000	0.000	0.001
				**Site 3**	0.000	0.000	–	0.000	0.001
				**Site 4**	−17.392	−12.714	−12.602	–	0.000
				**Site 5**	−5.054	−3.853	−3.823	9.731	–
**Unconsolidated (soft)**	>>> Coral Rubble	104.60	<0.001	**Site 1**	–	1.000	0.021	0.000	0.000
				**Site 2**	0.853	–	0.015	0.022	0.000
				**Site 3**	−2.770	−2.919	–	0.000	0.000
				**Site 4**	4.788	2.712	6.036	–	0.003
				**Site 5**	8.133	5.452	8.587	3.416	–
	> Sand/ Mud (<2mm)	227.36	<0.001	**Site 1**	–	0.000	0.000	0.000	0.114
				**Site 2**	−5.402	–	0.000	0.000	0.006
				**Site 3**	9.237	11.779	–	0.017	0.000
				**Site 4**	7.956	10.814	−2.793	–	0.000
				**Site 5**	−2.066	3.165	−9.686	−8.410	–

CATAMI parent-classifications for each label are shown, with intermediary steps indicated by “>”. Site pairwise comparisons are calculated using Dunn’s Multiple Comparison post-hoc tests, with Z-scores displayed below the dotted lines and corrected p-values (Benjamini-Yekuteili) above. Positive Z-scores are indicative of greater column-header means, while row-headers are supported by negative values. Significant pairwise comparisons (α = 0.05) are highlighted with grey cell shading. Results for other parent classes are provided in S1 Table.

Coral morphology type and presence also varied significantly between sites (Kruskal-Wallis; Table 1; Fig 4B). Massive colony morphotypes were the most common morphotype in our surveys (24.2% cover) and were present at all sites. Within individual sites, massive coral colonies were the most common morphotype at Sites 1, 2, and 5 (49.2%, 61.9%, and 53% cover, respectively; Dunn’s; Table 1, Fig 4B). Tabulate growth was the second most dominant morphotype (20.9% cover across all sites), yet they were absent from Sites 1–3. This marks a significant divide between Sites 1–3 and Sites 4–5 (Kruskal-Wallis; Table 1) (Dunn’s; Table 1). Encrusting coral growth was comparatively rare across all sites (4.6% cover), except for Site 3 in which it was the dominant coral morphotype (59.6% cover; Fig 3B). In lieu of scleractinian cover, substantial macroalgal growth was detected in Sites 1–3 (Fig 4A). Percent cover of most macroalgae sub-categories were found to differ significantly between Sites 1–3 and Sites 4–5 (Dunn’s; S1 Table).

#### Benthic Site-Dissimilarity.

**Fig 5:**
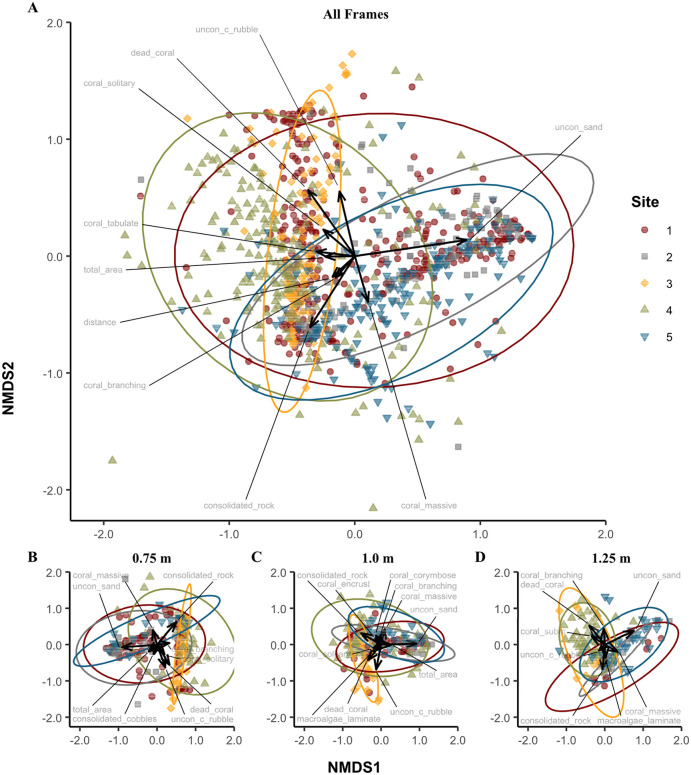
Two-dimensional non-metric multidimensional scaling (nMDS) of benthic compositional site dissimilarity (Bray-Curtis) within the Groote Archipelago. Site-clusters utilise CATAMI classifications and are visualised through elliptical multivariate t-distributions and distinguished by colour. Statistically (α = 0.05) and ecologically significant (r^2^ ≥ 0.05) multiple regression vectors are overlaid to highlight the strongest drivers of ordination shape. To isolate location effects from potential frame-distance interference, ordination was calculated using frame-subsets of: (A) all frames (stress = 0.206); (B) 0.75 m (stress = 0.163); (C) 1.0 m (stress = 0.192); and (D) 1.25 m (stress = 0.192) above the benthos.

Bray-Curtis dissimilarity matrices revealed high benthic compositional variability between frames, as seen by moderate stress of global nMDS ordination (0.206; Fig 5A). Elliptical groupings highlighted two moderately distinct site-clusters: Sites 2 and 5; and Sites 3 and 4; with Site 1 as an intermediate (Fig 5A). Vector analysis highlighted that, while significant, frame-benthos distance only had minor influence on global nMDS ordination (r^2^ = 0.063, p = 0.0001). Unconsolidated sand was the most influential benthic category (r^2^ = 0.809, p = 0.0001), driving the clustering of Sites 2 and 5. Numerous benthic categories influenced the clustering of Sites 3 and 4, with bedrock (r^2^ = 0.497, p = 0.0001) and dead coral (r^2^ = 0.461, p = 0.0001) among the most influential). Only three distance categories (0.75, 1.0, and 1.25 m) had sufficient representation across all sites (>30 frames per site) to perform subset analyses, yet results largely corroborated global analysis: Sites 2 and 5; and Sites 3 and 4 remained distinct, however Site 1 had lower dispersion and now clustered with the Sites 2 and 5 (Fig 5B-D). Vector analyses were similarly consistent with global ordination, with unconsolidated sand driving the clustering of Sites 2 and 5, while bedrock and dead coral led the clustering of Sites 3 and 4 in all subsets (Fig 5B-D). Consequently, individual frames were deemed compositionally informative, despite their inconsistent area.

PERMANOVA was deemed inappropriate for multivariate site comparison as frame dispersion was significant (PERMDISP; F = 16.4, df = 4, p < 0.0001). ANOSIM was employed instead, as it makes no assumptions about dispersion [63]. ANOSIM confirmed nMDS analyses, indicating an overall benthic dissimilarity between sites of 15.2% (p = 0.0001). Site pairwise dissimilarities were far greater, ranging from 66.0% (Site 1 compared to Site 2) and 78.7% (Site 2 compared to Site 4; SIMPER; S2 Table). Across all comparisons, between eight and twelve CATAMI classifications drove >90% of benthic dissimilarity (S2 Table; SIMPER). Coral morphology was a key differentiator of benthic composition within many sites. This was particularly apparent between Sites 4 and 5, where massive and branched (*i.e.*, tabulate, corymbose, and branching) morphologies drove 32.4% of overall dissimilarity (S2 Table). Conversely, differences in substrate consolidation and coral mortality between Sites 4 and 5 were both found non-significant (S2 Table). Comparisons between Sites 1 and 2 were largely the inverse of Sites 4 and 5; substrate composition drove most site dissimilarity (54.8%; SIMPER; S2 Table), though mortality and rubble was similarly insignificant. Coral rubble, and to a lesser extent mortality, were however, important drivers of benthic dissimilarity between all Site 3 comparisons (SIMPER; S2 Table). Notably, these benthic categories accounted for a cumulative 30.4% and 28.1% (SIMPER) of Site 3 dissimilarity to Sites 4 and 5, respectively. Further, the lack of scleractinian coral morphological diversity and cover within Site 3 instigated much of the remaining dissimilarities to other sites (26.8% vs. Site 4; 17.6% vs. Site 5; SIMPER; S2 Table).

#### Benthic Site Indicators.

Key indicators of benthic composition for each site were identified through indicator species analysis (ISA). Thirty-five of the CATAMI classifications utilised within this study were found to be statistically significantly single- (n = 17) or multi-site indicators (n = 18; Table 2). Two classifications (“Stony Corals> Dead”, IV = 0.780; and “Consolidated (hard)> Rock”, IV = 0.923) were most strongly associated with all study sites and thus could not be assigned significance. Other consolidated classifications were similarly ubiquitous, associating weakly with Sites 1, 2, 3, and 5. Similarly, massive stony corals had strong association with all locations except Site 3 (IV = 0.701). Unconsolidated features (*i.e.*, sand) were generally powerful indicators of Sites 1, 2, and 5. Consistent with high intra-site dispersion observed within nMDS ordination, Site 1 did not have any single-site indicators.

**Table 2:** Statistically significant indicators of study site benthic composition, as determined through Indicator Species Analysis (ISA).

CATAMI Classification		Strength				Association				
**Parent**	**Class**	**A**	**B**	**IV**	**p-value**	**Site 1**	**Site 2**	**Site 3**	**Site 4**	**Site 5**
**Black & Octocorals**	> Blue Coral (Heliopora)	0.998	0.419	0.647	0.005				**•**	
	> Branching (3D)	0.600	0.066	0.199	0.005					**•**
	> Massive Soft Corals	0.902	0.114	0.320	0.005					**•**
	> Whip	1.000	0.036	0.190	0.005					**•**
**Cnidaria**	> Hydroids	1.000	0.030	0.173	0.005					**•**
	> True Anemones	0.987	0.018	0.171	0.035		**•**			**•**
**Consolidated (hard)**	> Boulders	0.946	0.111	0.323	0.010	**•**	**•**	**•**		**•**
	> Cobbles	0.994	0.098	0.312	0.005	**•**	**•**	**•**		**•**
**Echinoderms**	> Sea Cucumbers	0.937	0.035	0.181	0.005		**•**		**•**	**•**
	> Sea Urchins	1.000	0.162	0.402	0.005					
**Macroalgae**	> Articulated Calcareous	1.000	0.063	0.251	0.005			**•**		
	> Erect Coarse Branching	0.994	0.140	0.374	0.005		**•**			
	> Erect Fine Branching	0.907	0.270	0.495	0.005			**•**		
	> Filamentous/ Filiform	1.000	0.061	0.248	0.005		**•**			
	> Globose/ Saccate	1.000	0.018	0.132	0.025		**•**			
	> Laminate	0.994	0.306	0.552	0.005	**•**	**•**	**•**		
	> Sheet-like/ Membranous	0.798	0.289	0.481	0.005		**•**			
**Molluscs**	> Bivalves	0.829	0.349	0.538	0.005	**•**				**•**
**Seagrasses**	> Elliptical Leaves	1.000	0.061	0.248	0.005		**•**			
**Sponges**	>> Encrusting	0.850	0.175	0.386	0.005	**•**	**•**	**•**		
	> Erect Forms	0.904	0.040	0.190	0.025	**•**	**•**			
	> Massive Forms	0.967	0.066	0.253	0.005	**•**	**•**			
**Stony Corals**	> Branching	0.974	0.260	0.503	0.005				**•**	**•**
	> Corymbose	0.982	0.555	0.738	0.005				**•**	**•**
	> Digitate	0.780	0.405	0.562	0.005				**•**	
	> Encrusting	0.935	0.269	0.501	0.005		**•**	**•**	**•**	**•**
	> Foliose/ Plate	0.930	0.084	0.279	0.005		**•**		**•**	**•**
	> Massive	0.957	0.508	0.701	0.005	**•**	**•**		**•**	**•**
	> Solitary	0.943	0.369	0.590	0.005	**•**		**•**	**•**	
	> Staghorn	0.987	0.018	0.133	0.025					**•**
	> Sub-massive	0.645	0.509	0.573	0.005				**•**	
	> Tabulate	0.818	0.509	0.645	0.005				**•**	
**Unconsolidated (soft)**	>>> Barnacle Plates	0.876	0.054	0.218	0.010	**•**	**•**			
	>>> Coral Rubble	0.928	0.535	0.705	0.005	**•**	**•**	**•**		
	> Sand/ Mud (<2mm)	0.942	0.585	0.742	0.005	**•**	**•**			**•**

High coral rubble (IV = 0.705) and macroalgae cover were defining features of Sites 1–3. All macroalgal sub-classifications were associated with one or more of these sites, with laminate growth prevalent in all three (IV = 0.552). Conversely, scleractinian coral cover was more frequently associated with Sites 4 and 5. Corroborating SIMPER analyses found branched coral morphology was associated primarily with Site 4; tabulate (IV = 0.645), sub-massive (IV = 0.573), and digitate (IV = 0.562) growth forms were all found as powerful single-site benthic indicators of Site 4. While Site 5 shared corymbose (IV = 0.738) and branching (IV = 0.503) corals as multi-site indicators with Site 4, soft coral classifications dominated its single-site indicators.

### Sea Surface Temperature

**Fig 6:**
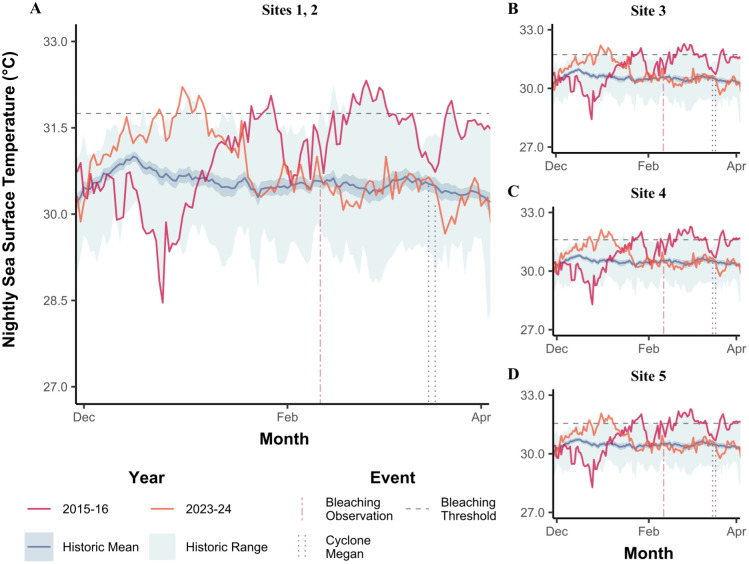
Satellite-derived nightly sea surface temperature (SST; °C) from the study sites within the Groote Archipelago. As obtained by the NOAA *CoralTemp* SST Daily Global 5 km Satellite v3.1 for: (A) Sites 1 and 2 (same data covers both sites as they are too close together to have unique datasets); (B) Site 3; (C) Site 4; and (D) Site 5. SST readings from the study period (1 June 2023–31 May 2024; orange) are contrasted with twenty-year historic means (dark blue; 2003–2004 to 2022–2023). Shaded areas denote standard error (dark blue) and range extrema (light blue). Individual bleaching thresholds are highlighted for each site (horizontal dashed line; black), calculated using maximum monthly means + 1.0 °C [(A) 30.75 °C, (B) 30.73 °C, (C) 30.60 °C, and (D) 30.56 °C]. For comparison, SST readings from the summer of 2015–16, also a global bleaching event year, (red) are shown. Initial observations of bleaching in the area are indicated by vertical dashed lines (pink; 11 Feb 2024), while dual vertical dotted lines (purple) indicate the subsequent passage of Cyclone Megan (Cat. 4, 16–18 Mar 2024). Data for Sites 1 and 2 (A) has been enlarged for increased readability as SST patterns were largely similar for all sites.

Nightly SST readings obtained from 1st June 2003 – 31st May 2024 ranged from 20.70–32.32 °C. The overall mean temperature was 28.0 ± 0.02 °C, while summer and winter means were 30.5 ± 0.01 °C and 24.4 ± 0.3 °C, respectively. This seasonal variation was significant according to ANOVA (F = 51311.09, df = 11, p < 0.0001) and average temperature varied significantly between sites (F = 3.77, df = 3, p = 0.0101). Seasonality was found to have a moderating effect on this relationship (F = 9.79, df = 33, p < 0.0001). Pairwise post-hoc analysis highlighted two distinct site clusters: Sites 1 and 2, (Fig 6A) and Sites 4 and 5 (Fig 6C-D), with Site 3 acting as an intermediate (Fig 6B). Site 4 had slightly cooler summer temperatures compared to other sites (−0.09 °C vs. Sites 1 and 2, p = 0.0414) and Site 5 (−0.12 °C vs. Sites 1 and 2, p = 0.0012; −0.10 °C vs. Site 3, p = 0.0088) and slightly warmer winter temperatures (+0.21 °C vs. Sites 1 and 2, p = 0.0166; + 0.18 °C vs. Site 3, p = 0.0519).

Overall mean SST varied significantly both seasonally (*i.e.*, by month) and annually (F = 558.41, df = 20, p < 0.0001). There was strong evidence of an interaction effect (F = 123.61, df = 220, p < 0.0001), with post-hoc analysis revealing mean SST during the summer of 2023−24 did not differ significantly from other years of globally intense thermal stress (vs. 2015−16, p = 0.8205; vs. 2019−20, p = 0.4077; vs. 2021−22, p = 0.997). Moreover, substantial heating was highlighted in December 2023 (+0.39 °C, p < 0.0063) and January 2024 (+1.22° C, p < 0.0001) compared with the year prior. Across all sites, maximum monthly means occurred in December, ranging from 30.74 °C (Sites 1 and 2) to 30.55 °C (Site 5). From December 2023 to January 2024, SST was observed at all sites to exceed the bleaching threshold on two separate occasions (Fig 6). At Site 4, bleaching thresholds were exceeded for 12 days total (10 for Sites 1−3, and 5), with 10 of those days being consecutive (9 consecutive in Sites 1−3, and 5); culminating in a DHW value of 2.10 (Sites 1 and 2, 1.71; Site 3, 1.74; and Site 5, 1.79). As such, all sites were categorised under the stress level of “Bleaching Warning”. Thermal stress had largely subsided prior to any observations of bleaching, with mean temperatures during February being notably cooler than in comparative years (−1.02 °C vs. 2015−16, p < 0.0001; −0.79 °C vs. 2019−20, p < 0.0001). Correspondingly, while SST did decrease in the immediate aftermath of Cyclone Megan, this effect was neither long-lasting nor outside of normal variability.

## Discussion

Here we present a study documenting benthic structure and composition at five key reef sites of the Groote Archipelago, as identified by the local Anindilyakwa people. This project aimed to provide a baseline of ecosystem health at present-day, with particular focus on coral cover and morphological diversity to support part of the Sea Country Management Plan (SCMP) for Groote Eylandt. All sites had scleractinian coral cover, however there were noticeable differences in both coverage and composition of key morphotypes. Primarily, branched morphologies – often key drivers of ecosystem functionality on traditional reefs [64] – were all but absent within Sites 1, 2, and 3 (Fig 4B). The rugosity of branching colonies provides crucial protection to reef taxa across all life stages [65,66], yet it is unclear how their low abundance at Sites 1–3 has affected overall reef function or what the drivers behind their absence at these sites are.

The lack of branching species at these sites suggests that environmental conditions may be too stressful or variable to support these generally sensitive species, or that previous stress events (bleaching events, storms, etc.) have eliminated them from the area – indicating that Groote’s reefs may be considered marginal reefs. Low structural complexity is a frequently described phenomenon in marginal reefs worldwide [67–69]. Without branching morphotypes, coral morphology in such systems is often dominated by massive coral morphologies – as is evident at Sites 1, 2, and 5. Challenging oceanographic conditions in marginal reef systems, such as high turbidity, temperature, or nutrient content, can create uniquely suboptimal ecosystems and challenge the paradigm of where corals can survive [67–69]. The inherent fragility of branched morphologies commonly precludes their development in marginal reefs, whereas the stress-resilient nature of massive corals is well-documented [70,71]. Beyond the coral community, fish assemblage functional and biological diversity in marginal reefs is also likely to be lower than more optimal or rugose reefs [72]. Yet, this lack of rugosity can result in more generalised and, consequently, robust assemblages to climatic variability or coral decline [69,73]. Despite significant work to date by the ALS Rangers and collaborators to document fish diversity within the archipelago [74], future work should consider assessing population changes at the differing sites, to better resolve any potential ecological consequences that may arise from the absence of rugose coral morphotypes. Further, *in situ* monitoring of environmental parameters such as temperature, turbidity, salinity, pH, flow and/or dissolved oxygen may provide additional context for these observations and confirm if these reefs may be considered “extreme” or “marginal” [47].

Despite the current scarcity of living branching coral morphologies, there was visual evidence of previous branching growth within many study sites, evidenced by the presence of dead branching coral skeletons. The intact nature of the branching framework and visible corallites suggested recent mortality [75]. While it is impossible to determine precise dates of mortality, rapid turfing algae growth consistent with the literature [76] points to the 2024 bleaching and/or subsequent cyclone likely being responsible for this loss of branching morphologies. Thus, it is possible that the absence of living branching colonies at Sites 1–3 could have been driven by recent, acute events, rather than ongoing marginal conditions. However, with the current dataset, it is not possible to be certain of this. Notably, tabular mortality was detected within Site 3, with no living functionally comparable alternative present (Fig 4B). Due to their lateral growth, tabulate corals can harbour fish of greater number and size than other morphotypes and their disappearance will likely have profound ecological ramifications [17,64]. This, combined with compositional similarity with Site 4 (Fig 5), perhaps indicates conditions within Site 3 were once different. Notably, previously unknown submerged reefs (at depths > 20 m) have been observed in other regions of the Gulf of Carpentaria [29,77] and were mainly dominated by foliose or tabular morphology corals (*Turbinaria* sp. and *Leptoseris* sp.), suggesting that these morphologies may be well-suited for this region. Summaries from larger expeditions across the Northern Territory further confirm the dominance of foliose and tabular morphology hard corals based on dredging and limited diving activities [29,30,77,78]. These reports reveal hard coral cover to be plentiful throughout the Northern Territory, but whether reefs are dominated by hard corals (>50% cover) like Site 4 is sparingly documented [79]. Surveys of deeper water shoals in the Arafura and Timor Seas, with some exceptions, have typically found hard coral cover to be 20–25% [79–81]. These findings suggest that assessments of a “healthy” reef in this region may differ from the definition of a “healthy” reef in areas like the Great Barrier Reef or the Indo Pacific (i.e., presence of branching species may not be key indicators of reef health at Groote Eylandt). However, without historical baseline knowledge of reef assemblages in this area, more data across additional reef sites, particularly coastal, shallow sites, is needed to confirm this.

Nightly SST readings reached 21-year historic highs from December 2023 to January 2024, staying >1 °C above the MMM for as many as 12 days (Fig 6). Per NOAA Coral Reef Watch methodologies, all sites bar Site 4 (2.1 °C-weeks) had Degree Heating Week (DHW) values that fell shy of 2 °C-weeks [56]. DHW values below 4.0 °C-weeks are generally considered insufficient to induce reef-wide bleaching, and mortality of heat-sensitive species only becomes expected at ≥8.0 °C-weeks [56,82,83]. Despite this, widespread bleaching (>62%) was evident within study sites. While there is wide expectation that marginal reefs, like those around Groote Eylandt, are more thermally resilient than other reefs due to inhabiting extreme environments [47,67], there is also precedence the opposite may be true – other marginal reefs have been observed to more readily undergo phase shifts during marine heatwaves [84–86]. It is possible that coral assemblages here already live on the limit of their thermal tolerance [47], though reef mean SSTs are far from global highs [87]. As bleaching is thought to be a mechanism of oxidative stress avoidance [88], water-flow can moderate the thermal tolerance of coral species [89]. It could thus be expected that corals within the Gulf of Carpentaria would more readily bleach due to the long residence time of water in the region. It should be noted that the NOAA’s Gulf of Carpentaria Virtual Station, the closest to Groote Eylandt, did report a DHW value of 3.9 °C-weeks on 13th January 2024 – just under the threshold for Bleach Alert Level 1. However, the Virtual Station product uses blended SST data from all regional reefs and is intended to signal users to look closer at finer spatial resolution analyses. Importantly, Groote Eylandt’s northern reefs are yet to be incorporated into any virtual station and are thus not accurately represented by this product. Regardless, it is clear the level of stress experienced within study sites exceeded expectations, with the observations reported here highlighting the importance of *in situ* data collection to validate predictive modelling.

It is not definitively known if Sites 1–3 exhibited bleaching as bleaching surveys did not extend to their locations. It is, thus, difficult to assess thermal and cyclonic stressors in isolation. While Site 5 experienced similar thermal stress to other sites, it was clear that stress event impact was relatively minimal. Massive and encrusting morphologies, dominant in Site 5, are capable of more efficient heat dispersal than branched colonies [90]. Similarly, shearing forces experienced during intense wave and current dynamics are more evenly dispersed across their compact surface [91]. Correspondingly, it is to be expected that coral mortality was proportionally greater within Site 4, a reef dominated by branching, corymbose, and tabulate colonies, which are typically considered to be susceptible to stress and wave damage [92]. Mortality within Site 3 was greater still, and while thorough oceanographic assessment of each site is beyond the scope of this paper, cold water through-currents felt during fieldwork in Site 4 may have alleviated thermal stress [93]. If Sites 3 and 4 truly contained similar coral assemblages prior to stress, this insulation may have driven their differing benthic compositions following a stress event. It remains unclear whether these ecosystems will recover without intervention, though natural phase shift reversals may be more common than previously thought [1]. Ultimately, long-term monitoring will be essential to understand the changes across all sites.

The inability to answer key questions about reef health, stress patterns, and recovery on Groote Eylandt’s reefs underscores the importance of formal long-term records. Given the lack of bleaching observed within Sites 4 and 5 during the June 2024 surveys, reef prognosis following the early 2024 stress events is likely positive – though the same cannot be assumed of the other sites. Future surveys will be required to determine if the baselines reported here represent the natural states of Sites 1–3, or if benthic composition at these reefs were shifting at the time of data collection. Frame-area variance between and within benthic surveys may limit comparative ability, though area-standardisation methodologies used here showed clear site differences. These methodologies provide novel advancement to inconsistent or disrupted benthic video analysis at medium-scale, allowing the establishment of benthic composition baselines on other reefs with minimal operator training. The selected study sites presented here only represent a relatively small area within Groote Eylandt, with many reefs in this region remaining undocumented. While findings from this study will directly influence ongoing development of the Sea Country Management Plan, more locations will need to be assessed and over longer temporal scales to truly assess the state of Groote Eylandt’s reefs. Baselines provided here represent the first of their kind within the Gulf of Carpentaria, and provide evidence of changing reef conditions on Anindilyakwa Sea Country. We acknowledge that our data was collected following a stress event, and thus may not represent a “healthy” baseline on these reefs. However, knowledge of the current state of the reef will be valuable for tracking recovery and change to support existing and future conservation and management efforts [94].

Ultimately, our study findings represent a necessary step forward for marine conservation within the Groote Archipelago. Benthic compositional baselines were determined for five key reefs, with scleractinian coral cover documented throughout. Furthermore, we document here the first known instance of mass coral bleaching within the Gulf of Carpentaria, challenging the perceived resistance of marginal reef systems to thermal stress. Yet, the absence of historical baselines underpins a need for increased scientific effort at this unique and under-studied site. Remote reefs, like those around Groote Eylandt, have all-too commonly been relegated as conservational afterthoughts, despite their immense importance to local communities. With the intensity and frequency of bleaching events globally only predicted to increase, so too must the effort and global parity of conservational research.

## Supporting information

S1 Fig Australian Reef Publications by Region. Number of publications conducted within major Australian Reefs since 1980 by region, as revealed by a systematic search of the ISI Web of Science database. Search conducted on 13/03/2024.
(TIFF)

S2 Fig Screenshot of BIIGLE Image Processing. Example screenshot of annotation and segmentation using the BIIGLE tool for a frame captured during a video survey.
(TIFF)

S1 Table Kruskal-Wallis H statistic and corresponding p-value for mean frame benthic composition of each study site. CATAMI parent-classifications for each label are shown, with intermediary steps indicated by “>”. Site pairwise comparisons are calculated using Dunn’s Multiple Comparison post-hoc tests, with Z-scores displayed below the dotted lines and corrected p-values (Benjamini-Yekuteili) above. Positive Z-scores are indicative of greater column-header means, while row-headers are supported by negative values. Significant pairwise comparisons (α = 0.05) are highlighted with grey cell shading. All classes shown in this table, as an expansion to Table 1.
(XLSX)

S2 Table Primary drivers of benthic compositional dissimilarity (Bray-Curtis) between sites, as categorised by the CATAMI classification scheme. Individual frame-contributions to overall site dissimilarity were calculated using permutational (n = 9999) SIMPER and shown as: average (avg.) contribution ± standard deviation; cumulative (cum.) proportional contribution; and permutational significance (p-value). Classifications are listed until at least 90% of overall dissimilarity is accounted for. Presented for various Site-Site combinations in different tabs of spreadsheet.
(XLSX)

S1 Dataset Raw benthic compositional data of each frame. Note: “label_names” are not unique (e.g., “encrusting” may refer to stony corals or sponges), with “label_ids” their unique identifier, instead. For full annotation schema, see “https://doi.org/10.5281/zenodo.3374162”.
(CSV)
